# Patient and Provider Perspectives on Cesarean Delivery Pain and Anesthesia Experiences: A Qualitative Study

**DOI:** 10.1177/26884844251364123

**Published:** 2025-08-01

**Authors:** Yunseo Linda Park, Briana Clifton, Rida Ashraf, Rose Barlow, Alexandra Anderson, Valeria Altamirano, Emily Miller, Mark Neuman, Grace Lim

**Affiliations:** ^1^ Department of Anesthesiology and Perioperative Medicine, University of Pittsburgh School of Medicine, Pittsburgh, Pennsylvania, USA.; ^2^ Brown University, Women and Infant’s Hospital, Providence, Rhode Island, USA.; ^3^ Department of Anesthesiology and Critical Care, University of Pennsylvania Perelman School of Medicine, Philadelphia, Pennsylvania, USA.

**Keywords:** analgesia, anesthesia, cesarean, patient experience, respectful maternity care, shared decision-making

## Abstract

**Background:**

There is a lack of evidence on the importance of pain or other aspects of clinical care in the overall patient experience and patient-centered outcomes in cesarean delivery. The purpose of this study was to discover patient priorities in cesarean delivery anesthesia experience, to compare patient and provider perspectives, and to explore attitudes on shared decision-making around anesthesia choices for cesarean delivery.

**Methods:**

Patients with recent cesarean deliveries and clinical care providers were approached using a purposeful sampling strategy for this prospective observational qualitative study. Patients were included if they were in the hospital within 72 hours of a cesarean delivery (scheduled or unscheduled), spoke English fluently, and had term gestation. Providers were included if they currently provide regular clinical care to patients having cesarean deliveries and have at least 3 years of practice experience. Semi-structured interviews were conducted using an interview guide. Interview transcripts were independently coded by three coders and qualitatively analyzed for major themes until thematic saturation was achieved.

**Results:**

A total of 42 participants (20 patients and 22 providers) completed interviews. Five major themes emerged reflecting patient attitudes and beliefs toward cesarean delivery experience: (1) effective communication, education, and respect; (2) emotional support by care team; (3) intraoperative pain or discomfort; (4) varying acceptability around pain therapies; and (5) stigma surrounding cesarean delivery. Five major themes emerged reflecting provider attitudes and beliefs toward cesarean delivery priorities: (1) complexity of pain responses; (2) multiple pain control strategies; (3) effective communication during emergency cesarean delivery; (4) patient psychological well-being during cesarean delivery; and (5) barriers to observing the patients’ birth plans.

**Conclusion:**

Patients and providers alike desire pain management, psychological well-being, and effective communication during cesarean delivery. Patients emphasize relationships and trust in their cesarean experience, while clinicians emphasize clinical complexities and physical treatments.

## Background

Pregnancy and childbirth are the most frequent reason for hospitalization in the United States, with greater than 30% of deliveries occurring *via* cesarean.^1^ The need to support individualized birth experiences is widely acknowledged,^2–4^ yet there is a lack of evidence on patient-centered priorities in cesarean birth experiences, particularly regarding anesthesia-related elements.

In North American and other high-resource settings, cesarean delivery typically involves neuraxial anesthesia, allowing the patient to remain conscious during the surgery. While this approach enables the patient and their support person to participate in the delivery and remember the birth event, it can also lead to apprehension about intraoperative physical sensations and side effects. Neuraxial anesthesia is considered the gold standard for cesarean delivery, because it provides more effective postoperative pain relief than general anesthesia and eliminates the need for airway manipulation, reducing the risk of failed airway interventions or aspiration events.^4,5^ However, some patients may prioritize other factors over these outcomes.^6^ Additionally, neuraxial anesthesia can occasionally fail to provide adequate anesthesia, with estimates ranging from 0.5% to 4% for spinal anesthesia and from 4% to 13% for epidural anesthesia in cesarean deliveries.^7–9^

Research indicates that patients highly prioritize avoiding intraoperative pain during cesarean delivery,^10^ but data on the relative importance of other patient-centered outcomes unrelated to the physical nature of the birth experience are lacking. Factors such as active participation in decision-making, maintaining a sense of control, and effective communication from care providers significantly impact patient satisfaction with the birth experience.^11–16^ Additionally, no studies have compared patient and provider perspectives on prioritizing aspects to optimize the cesarean delivery experience for the patient. Input from anesthesia clinicians is essential to identify perceived barriers and facilitators in achieving these patient-centered goals. It is crucial to clearly define patient-centered outcomes and clinician perspectives, particularly regarding anesthesia management for cesarean deliveries.

The purpose of this study was to explore and compare patient and provider perspectives on various aspects of anesthesia care during a cesarean delivery. Our primary questions were: (1) What are pregnant people’s wishes regarding their anesthesia experiences during cesarean delivery? (2) How important do patients perceive intraoperative and postoperative pain to be in their overall cesarean delivery experience compared to other factors? (3) How do these patient perspectives align with those of providers?

## Methods

The study was approved by the University of Pittsburgh Institutional Review Board (STUDY23060017). This prospective qualitative study included semistructured interviews of two study populations: patients who underwent cesarean deliveries, and clinical providers (anesthesiology, obstetricians, midwives, and nursing) who care for patients undergoing a cesarean delivery.

### Participants and recruitment

We used purposive sampling to recruit maternity care providers, aiming for a diverse range of professional roles and practice experiences. Recruitment was undertaken at two urban, tertiary academic maternity centers at the University of Pittsburgh Medical Center Magee-Women’s Hospital and University of Pennsylvania, to support direct interviews with research staff. The intention of purposive sampling was to recruit specific types of providers, that is, nursing staff from labor and delivery units, practicing obstetricians and anesthesiologists at attending, fellow, and resident levels, and midwives who provide direct care. Providers were recruited from both institutions, and patients were recruited from University of Pittsburgh only. For patient recruitment, all eligible postpartum patients at the study hospital were screened within 72 hours of cesarean delivery. Eligibility criteria included age ≥18, term gestation, English fluency, and capacity to provide informed consent. Patients were approached in person by trained research staff using a standardized script to minimize recruitment bias. Although some convenience elements influenced patient enrollment (*e.g.,* timing and availability for interview), this approach supported thematic saturation within a qualitative, exploratory framework.

### Enrollment procedures

Patients were eligible if they had a cesarean delivery. Patients in inpatient postpartum units were screened by mode of delivery. Patients who had a cesarean delivery at term gestation within the previous 72 hours were approached in person. Participants were excluded if they were unable to participate fully in the informed consent discussions or the interview for any reason or did not speak English fluently as the interviews were conducted in English language.

Providers were recruited *via* email or direct in person approach. Providers were included if they were an anesthesia provider, labor and delivery nurse, obstetrician, or midwife, who have cared for pregnant people undergoing cesarean delivery. Participants were excluded if they were unable to participate fully in the informed consent discussions or the interview. Informed consent was obtained from all participants.

### Interview methodology

A semi-structured interview guide (Supplementary Data S1) was developed to direct a discussion about what pregnant people prioritize about their cesarean delivery experiences, how important they perceive intraoperative and postoperative pain in their overall cesarean delivery experience compared with other factors, and how they perceived the process of shared decision-making with their clinicians on treatment of their pain or discomfort. The interview guide was developed by one investigator (G.L.) and was based on the conceptual framework by Patton.^17^ A similar guide was utilized and tested in a prior study.^18^ Patient interview questions addressed overall priorities and preferences in a cesarean delivery experience, expectations about cesarean pain and its management, respectful maternity care, shared decision-making, and emotional support. Provider interview questions addressed their priorities when caring for a patient undergoing cesarean delivery, pain management and education, respectful maternity care practices, shared decision-making efforts, and emotional support. Interviews were conducted privately, either in-person or *via* Microsoft Teams based on participant preference. Interviews were recorded, deidentified, and transcribed for coding purposes.

### Interview process and data collection

Written informed consent was acquired upon enrollment of the study subject. If the participant was virtually enrolled, verbal informed consent was acquired at the beginning of the interview. Microsoft Teams was used to both record and assist with transcription of the interview. Transcripts were subsequently reviewed and edited by investigators for accuracy. Participants were reminded that they could stop the interview at any time if they felt uncomfortable in answering any questions. Interviews were conducted by two investigators (Y.L.P. and B.C.) who were trained in qualitative research methods and used the semistructured interview guide-to-guide discussion (Supplementary Data S1). Recruitment of new participants continued until thematic saturation—the point where no new information was heard on subsequent interviews—was achieved for each participant group.

### Survey instruments and medical record data collection

A single demographic survey was completed by patients, and patients’ medical record data were collected by investigators. Data collected from the medical record included reason for cesarean, anesthesia type, total surgery time, postpartum hemorrhage status, estimated blood loss, use of sedative or opioid medications intraoperatively, body mass index, gravidity, parity, number of prior cesarean deliveries, opioid use disorder or substance use disorder with or without treatment, labor prior to cesarean, neonatal Apgar at 1 and 5 minutes, neonatal sex, and need for neonatal intensive care unit admission. Providers completed an intake survey including practice type and number of years in clinical practice.

### Qualitative data coding and data analysis

Qualitative coding software Atlas.ti (ATLAS.ti Scientific Software Development GmbH, Berlin, Germany, 1989–1992) was utilized. Initially, each transcript was individually reviewed by three investigators (Y.L.P., B.C., and R.A.) to identify preliminary codes. Then, the three coders convened to discuss, analyze, and reach an agreement on corresponding codes for specific words, phrases, and sentences of the transcripts. As such, an iterative content analysis process occurred, and the coding teams met to arbitrate differences in codes. The number of new codes that arose in each transcript was noted to evaluate for thematic saturation. Once no new themes arose, thematic saturation was declared as reached. Codes were then collated into potential themes, and quotes from transcripts were reviewed for selection to illustrate the themes. All coders played a role in theme analysis to explore any alternative interpretations. A secondary analysis explored differences in themes by provider role, and in themes by patients receiving scheduled or unscheduled cesarean delivery. Demographic data and medical record data were summarized using descriptive statistics including mean with standard deviation, median with interquartile range, and frequencies with percentages. All analyses were conducted using SPSS software version 28.0.1.1 (IBM).

## Results

A total of 20 patients and 22 providers participated in the interviews (Table 1). Ten (10/22, 45%) were anesthesia providers. Type of anesthesia was primarily spinal and the reason for having a cesarean were primarily an elective repeat cesarean delivery. Obstetric and neonatal outcomes are enumerated in Table 2. Key themes that emerged from patient and provider interviews are summarized in Table 3 (detailed table of quotations is available in Supplementary Data S2).

**Table 1.** Study Population Characteristics

Patient characteristics	Results (*N* = 20)
Age (years)	30 (2)
BMI (kg/m^2^)	38 (6)
Gravidity	2 (1–3)
Parity	1.5 (1–2)
Number of prior cesarean deliveries	0 (0–1)
Mental health history	
Anxiety or depression	12 (60%)
Other mental illness	3 (15%)
Racial identity	
White	14 (70%)
African American	4 (20%)
Asian	2 (10%)
Education level	
Some high school, no diploma	1 (5%)
High school diploma	5 (25%)
Bachelor’s degree	11 (55%)
Master’s degree	1 (5%)
Doctorate degree	2 (10%)
Average household income	
Less than 50,000	6 (30%)
50,000–100,000	4 (20%)
100,000–150,000	5 (25%)
150,000 or more	5 (25%)
Anesthesia type	
Spinal	13 (65%)
Epidural	9 (45%)
General anesthesia	0 (0%)
Other	0 (0%)
Reason for cesarean delivery^a^	
Arrest of descent/dilation	2 (9%)
Nonreassuring fetal status	5 (23%)
Elective repeat	6 (27%)
Malpresentation	5 (23%)
Other	4 (18%)

Provider characteristics	Results (*N* = 22)	Average years in practice
Anesthesiologist	5 (23%)	14 (13)
CRNA	5 (23%)	13 (11)
Obstetrician	3 (14%)	10 (6)
Nurse	7 (32%)	8 (7)
Other^b^	2 (9%)	12 (10)

The population included patients and providers. Data are presented as mean (standard deviation), median (interquartile range), or frequency (percentage).

^a^ Some patients had more than one reason for cesarean delivery.

^b^ Other providers include PA-C (physician-assistant certified) and CNM (certified nursing midwife).

BMI, body mass index; CRNA, certified registered nurse anesthetist; no., number.

**Table 2.** Obstetric and Neonatal Outcomes

Labor and delivery outcomes	Result (*N* = 20)
Maternal outcomes	
Labor before cesarean (yes)	5 (25%)
Median total surgery time (minutes)	50 (42–68)
Postpartum hemorrhage (yes)	1 (5%)
Median estimated blood loss (mL)	583 (491–724)
Use of sedative or opioid medications intraoperatively (yes/no)	2 (10%)
Neonatal outcomes	
Apgar^a^ 1 minute	8 ± 2
Apgar^a^ 5 minutes	9 ± 1
Neonatal sex	
Male	9 (45%)
Female	11 (55%)
Need for NICU (yes/no)	3 (15%)

Data are presented as mean ± standard deviation, median [interquartile range], or frequency (percentage).

^a^ The Apgar score is a system used regularly on newborns right after birth (at 1 and 5 minutes) as a fast way for clinical teams to gauge the necessity and effectiveness of neonatal resuscitation. It evaluates aspects such as color, tone, grimace, pulse, and respiratory effort. Scores above 7 are normal; 4–6 are classified as low, and 3 or below signal critical levels requiring immediate resuscitative measures.

NICU, neonatal intensive care unit.

**Table 3.** Patient and Provider Themes. Themes Identified by Semi-structured Interviews with Patients Who Experienced Cesarean Delivery and Providers Who Provide Cesarean Delivery Clinical Care More Complete Example Quotations Can be found in Supplementary Data S2

Theme	Code	Example quotations
Patient perspectives		
Effective communication, education, and respect	Desire to understand backup plan or alternatives	“I’m a planner, I need to understand... I need to know what you’re going to do so I’m prepared for it mentally.”
	Importance of tailored education	“The things the providers say should be accessible to everyone.”
	Comfort from real-time updates	“Nurses were keeping me informed, calming me down… it was just more of a pleasant experience…”
	Perceived discrimination	“My weight… added complications… some doctors looked at me a little bit different…
Emotional support by care team	Effect of being awake during a major surgery	“Mentally, I think it’s a little bit more tough… you’re going into a surgery and you’re wide awake…”
	Importance of mutual respect	“Mutual respect is everything… [without it] I wouldn’t feel like you’re handling my concerns.”
	Comforting environment created by care team	“They just boosted me up… gave me the strength to go for the operation…”
Intraoperative pain or discomfort	Patient discomfort (but not in pain) during cesarean delivery	“It was just the tugging… it was getting too much…”
	Patient felt pain during cesarean delivery	“I was in a lot of pain… I felt every single amount of pain…”
	Inadequate intraoperative pain management	“They gave me more… but it was too late.”
	Lack of pain management discussion	“I don’t think they… talked to me about options. They told me I would be given this. I was under the assumption that’s exactly what is given.
	Fear of intraoperative pain	“It was a fear that what if the spinal doesn’t work…”
	Anesthesiologist addressing pain concerns	“I actually did experience pain… they were very like, if you’re feeling anything, tell us.”
Varying acceptability around pain therapies	Patient aversion to opioids	“I didn’t want any [opioids]… I don’t want to be taking care of a baby while I’m on a narcotic.”
	Concerns about anesthesia side effects	“When I hear side effects, I get more concerned…”
	Preference for general anesthesia	“I didn’t expect to feel all that amount of pain… I would’ve felt better being put under.”
	Prioritizing baby’s needs over pain management	“I would want whatever was best for my son to happen…”
Stigma surrounding cesarean delivery	Efforts or desire to mirror vaginal birth	“They were able to accommodate… what I had hoped for…”
	Social judgment around birth	““Women will make comparisons… both of those are real births…”
Provider perspectives		
Complexity of pain responses	Recognition of pain complexity	“Pain is not just about the medicines we’re giving… it’s very subjective.”
	Importance of individualized pain management	“One treatment does not fit all… you have to individualize your approach.”
Multiple pain control strategies	Patient education on pain management	“When patients have reasons for going beyond our standard therapy…”
	Importance of multimodal pain management.	“The patient is getting everything from that multi-modal arsenal…”
Effective communication during emergency cesarean delivery	Desire for better intraoperative pain education	“In an emergency c-section… they might not be quite as well informed…”
	Reducing patient anxiety through education	“A lot of people are anxious because of the unknown… being informed on step by step…”
	Balancing provider opinion with patient autonomy	“Almost always there’s more than one option… support whatever informed decision they make.”
Patient psychological well-being during cesarean delivery	Priority for respectful communication	“Patients may have had a traumatic past birth… just being respectful…”
	Recognition of patient trauma with general anesthesia	“…now there’s a time in their life, maybe arguably one of the most important times in their life, and they can’t remember it. That’s some real trauma.”
Barriers to observing the patients’ birth plans	Desire for flexibility in protocols	“Certain providers actually didn’t mind turning off the lights…”
	Bonding with newborn	“In some cases, a patient desires skin-to-skin contact during a c-section but may be vomiting throughout the procedure. This makes it challenging to facilitate that bonding moment, which is difficult for me as I want them to have the best experience possible.”
	Recognizing “beauty of birth” beyond medical aspects	“[our hospital] is very efficient… but for this patient, it’s their whole world. They’re birthing a child… So just be cognizant that you’re in a monumental moment in their life and act accordingly.”
	Challenges in accommodating birth preferences	“…Sometimes it can be a little bit frustrating because we don’t have the same values or views on those issues, and you’re in the tough spot…But being able to go beyond what your views are and saying, hey how can you try to make it happen? …Let’s try to respect those wishes.”
	Views on health care system stress	“People sometimes are… fixated on just getting through the day… they forget that that’s important.”

### Patient themes

#### Effective communication, education, and respect

Patients shared varied birth experiences highlighting the importance of communication and respect. Some patients reported negative interactions, such as feeling a lack of compassion from an anesthesiologist, feeling disrespected when their weight was discussed by an obstetrician, or sensing judgment from a nurse due to their age. While some patients experienced adequate and helpful communication with the anesthesia team when they felt pain or discomfort during their cesarean, others shared that there was insufficient communication to address their concerns.

Patients appreciated real-time updates throughout the surgery, which increased their comfort levels. They expressed a need for better preoperative education on backup plans and alternative options for unexpected situations in the operating room. Detailed preoperative discussions about potential scenarios and outcomes were perceived to be comforting, as they supported emotional preparation for emergencies. Notably, patients emphasized the importance of providers understanding their education and medical literacy levels to tailor conversations effectively. They valued ongoing inclusion and empowerment in decision-making throughout the delivery process.

Provider comparison: Providers similarly emphasized the importance of communication and education both before and during cesarean delivery, and they believed these elements enhance patient satisfaction with their cesarean experience.

#### Emotional support by care team

Many patients were satisfied with the emotional support they received from the care team. While it was difficult for some to comprehend that they would be awake during surgery, reassurance from providers helped addressed these concerns, making the experience more comfortable. Familiar faces in the care team as well as knowing the surgeon greatly increased patients’ comfort and trust, contributing to their sense of confidence in the care team and easing their anxiety when entering the operating room.

In contrast, when patients perceived a lack of emotional support, they felt unsafe and distrustful of the clinical team. For example, one patient felt her birth plan was ignored and unsafe around her clinicians. The awake status during surgery, coupled with a lack of coaching and emotional support, intensified her feelings of uncertainty and fear.

Provider comparison: Providers similarly recognized this monumental moment in patients’ lives and understood their role in shaping the experience positively or negatively. However, they did not specifically emphasize coaching or emotional support.

#### Intraoperative pain or discomfort

Some patients expected complete numbness from anesthesia during cesarean delivery and were surprised by the sensations they felt during surgery. Although most sensations were not sharp pain, the pressure, tugging, and moving of abdominal parts were unsettling and uncomfortable for many. Three patients reported feeling pain and discomfort during their cesarean delivery. One was dissatisfied with the care team’s efficiency and speed in managing her pain, while another felt that her concerns were adequately addressed by the anesthesiologist. These experiences contributed to their fear of choosing cesarean delivery in the future. Discussing pain management options and contingencies for discomfort prior to surgery provided a sense of comfort and relief.

Provider comparison: Intraoperative pain is a major provider concern for providers during cesarean deliveries. Providers emphasize the complexity of pain expressions and the importance of educating patients on the difference between pressure and sharp pain. However, one provider noted that even nonsharp sensations such as pressure can be perceived as painful and contribute to patient suffering.

#### Varying acceptability around pain therapies

Many patients were hesitant to use medications or opioids for pain control despite provider recommendations. Some felt that they did not need strong medications, while others feared developing an opioid addiction. Most patients were concerned that the side effects could hinder their ability to care for and bond with their newborn.

Patients also expressed concerns about anesthesia side effects such as headaches, swelling, shaking, and chronic migraines. One patient shared that they would have preferred general anesthesia to avoid the uncomfortable intraoperative sensations, even though they were not sharp pain.

Provider comparison: Providers valued multiple strategies for pain control, recognizing that no single therapy works equally for everyone. They perceived more psychological trauma associated with general anesthesia compared with neuraxial anesthesia and expressed hesitancy to use general anesthesia unless in an emergency. Providers emphasized the need to balance their recommendations with patient autonomy and acceptability when making treatment decisions.

#### Stigma surrounding cesarean delivery

Most patients voiced a preference for vaginal delivery over cesarean. They felt more in control of their birthing process when “choosing the natural path” and considered elements such as cord clamping, skin-to-skin, and breastfeeding, as critical parts of their birth experience. Patients were generally unaware that these elements could be incorporated into cesarean deliveries. They worried that the “medicalized” nature of cesarean delivery would detract from the birth experience and the “beauty of [their] baby being born.” One patient shared that she experienced judgment from “women in the community” who viewed vaginal delivery as a “real” birth, which contributes to the stigma surrounding cesarean deliveries.

Provider comparison: Providers similarly noted the perceived medicalization of the cesarean delivery process and emphasized the importance of recognizing the “beauty of birth” beyond the medical aspects. They observed that many patients preferred vaginal delivery and felt that education on the overall safety of cesarean delivery could help alleviate their concerns.

### Subgroup: Scheduled versus intrapartum cesarean

Among the 20 participants, 5 underwent cesarean birth after laboring, while 15 had scheduled cesarean deliveries. Participants with intrapartum cesareans described more acute emotional responses, including confusion, fear, and a lack of preparation. These individuals more frequently emphasized the distress of unexpected surgical intervention, the rapid progression of events, and the emotional toll of feeling excluded from decision-making during a perceived emergency. In contrast, those with scheduled cesareans more commonly discussed anticipatory anxiety and logistical challenges but were less likely to describe feelings of emotional trauma or loss of control. Several scheduled cesarean participants noted appreciation for preoperative counseling and structured planning.

### Provider themes

#### Complexity of pain responses

Providers emphasized the complexity of pain experience and pain expression: not only is pain felt and expressed differently between patients, but pain is also complicated by quality and intensity. As such, one standard approach to pain management does not fit all. Anesthesia providers highlighted the difference between pain and pressure. They stated that a clinical standard is that while pressure is considered a normal feeling during a cesarean delivery, sharp pain is not. Providers emphasized the importance of educating patients on this difference so that they can articulate whether a sensation is pain or pressure. However, one provider who gave birth *via* a cesarean herself pointed out that pressure can also be painful, and that these binary ideas can be a source of confusion for many patients.

Providers highlighted the importance of responding to patients when they express any type of intraoperative discomfort. They suggested responding by acknowledging and validating the patient’s concerns, followed by appropriate interventions. This approach aims to ensure patients feel heard and supported emotionally.

Providers stressed the need for continuous communication regarding pain during the procedure: alerting patients to moments of increased pressure and regularly checking with their comfort level can enhance their overall experience. Providers observed that patients often do not vocalize their discomfort, highlighting the importance of interpreting nonverbal cues to identify and address discomfort promptly.

Patient comparison: Many patients voiced that the pressure they felt during the procedure was quite uncomfortable and painful to them.

#### Multiple pain control strategies

Providers noted a need to leverage a variety of pain management modalities, such as transversus abdominis plane blocks, nonopioid pharmacologic options, and nonpharmacological options. It was emphasized that a multimodal approach is most effective, and providers should also be well-educated on nontraditional pain management options.

Providers compared general versus neuraxial anesthesia and what each option might mean for the patient’s experience. Some providers felt hesitant to go down the general anesthesia route due to safety concerns and more postoperative pain after general compared with neuraxial anesthesia. Providers also associated general anesthesia with a higher likelihood of traumatic experiences for the patient. Many scenarios where general anesthesia is employed have involved deteriorating patient physical stability, or intraoperative pain that fails to be resolved, the totality of which was observed to be interpreted as traumatic by their patients.

Providers noted that patients having emergent cesarean delivery often feel more pain, highlighting the relationships between heightened anxiety of a patient and their sensitivity to pain. They discussed urgent situations leading to heightened emotions and sensations, leading to differences in physical sensations.

Patient comparison: Patients reported widely varying levels of pain or discomfort throughout their cesarean delivery process and desired a broad arsenal of pain management modalities.

#### Effective communication during emergency cesarean delivery

Effective communication was a dominant theme throughout all interviews. Providers suggested earlier discussions, potentially during the prenatal care period, with patients regarding the possibility of an emergency cesarean delivery. This would ensure that patients are not left with a short window of time to make the decision to deliver *via* a cesarean and are better educated about what a cesarean experience entails. A key tactic providers mentioned was to frame discussions based on patient’s concerns and questions. Pausing and ensuring that patients are in understanding can help them approach the experience with less anxiety. Beyond informing patients about procedural details, providers also discussed the importance of explaining what the postpartum period entails. Due to the number of people that make up the care team of a patient, there was a perceived disconnect among providers regarding who is responsible for educating the patient on specific parts of the birth process. Although overwhelming a patient with too much information was undesirable, earlier discussions to set expectations and to codevelop goals and priorities were perceived as beneficial. Medical decisions were felt to need to strike balance between provider recommendation and patient autonomy.

Patient comparison: Patients similarly noted the positive impact of effective communication and education geared toward their level of education and understanding. Patients expressed feeling heard and respected when providers took time to fully educate them on the anticipated cesarean delivery experience.

#### Patient psychological well-being during cesarean delivery

Providers discussed their role in “supporting women during and after” a perceived complicated childbirth. They stressed the importance of being aware and protective of the patient’s intraoperative mental well-being and safety. They highlighted the need to be mindful when communicating with other providers during the procedure. One stated that although it may feel like any other routine day to the staff, the patient who is awake on an operating room table can be easily affected by what they hear. Providers also mentioned that patients may feel less autonomous or in control when undergoing a cesarean delivery compared with a vaginal delivery. They noted that patients having a cesarean delivery can benefit from the extra support from the staff.

Trauma-informed care was repeatedly mentioned by providers. Providers recognize the weight of their words and actions, especially when the patient may have had a previous traumatic delivery. Providers discussed the importance of understanding what may be triggering for the patient and that shaping their care around the patient’s psychological well-being can go a long way in preventing another negative experience.

Patient comparison: Patients similarly expressed benefiting from the emotional support providers provided. Many patients were fearful entering the operating room and believed that words of encouragement from providers helped to reduce their anxiety.

#### Barriers to observing the patients’ birth plans

All providers acknowledged the importance of accommodating the patient’s birth plan as much as possible but recognized practical limitations to reaching this goal for all patients. Although there are challenges when the patient undergoes an emergency cesarean delivery, providers discussed the possibility of increasing flexibility of protocols to better accommodate patient comfort. For example, one provider mentioned adjusting the lights in the operating room to create a calmer environment for the patient without interfering with the conditions of the surgical field. Providers noted there are ways to help the new parent bond with her newborn despite having gone through a cesarean. Provider gender preference also emerged as an issue that posed challenges to the provider team, especially when there may only be a male attending on shift. One provider mentioned overworked and burned-out health care workers lacking the flexibility or empathy to consider what might be important to the patient, citing room for systemic improvement. There was belief that if providers can overcome the challenge of maintaining sensitivity amid their routine procedures, they can better comprehend the significance of the moment for the patient.

Patient comparison: Patients feel highly respected and heard when providers are perceived to prioritize the accommodation of their birth plans. When not possible to accommodate, patients felt it was still important that they remained part of decision-making throughout all events.

### Subgroup: Differences by provider type

Although provider responses were originally analyzed as a single group, we conducted a secondary analysis to explore differences in themes by provider role. Although limited by sample size, we observed some notable patterns. First, anesthesiologists frequently focused on the technical aspects of pain management, highlighting the nuances of pressure versus pain and expressing concern for the emotional impact of failed neuraxial techniques. Their narratives often included strategies to prevent and respond to intraoperative discomfort. Second, nurses emphasized the emotional support they provide in the operating room, often describing their role in creating a calming environment, offering real-time updates, and reading nonverbal cues to detect distress. Finally, obstetricians described navigating the tension between clinical urgency and honoring patients’ birth plans. They frequently reflected on shared decision-making in emergent situations and acknowledged the emotional consequences of unplanned cesarean deliveries. These distinctions offer preliminary insight into the unique perspectives brought by different members of the care team and suggest the value of further role-specific analysis in future research.

## Discussion

The primary findings of the study reveal that both patients and providers prioritize not only pain management during cesarean delivery, but also emotional and psychological support. Both groups emphasized the importance of effective communication, education, and efforts to accommodate individualized birth plans. Yet, differences emerged regarding pain versus pressure expectations and the acceptability of therapies such as medications or general anesthesia. These findings help fill knowledge gaps about patient perspectives on cesarean delivery anesthesia, providing insights into future research surrounding patient preferences and improved patient-centered outcomes.

Our results are consistent with other studies emphasizing respectful maternity care and nonphysical aspects of a positive birth experience. Despite global efforts advocating for respectful maternity care,^19^ some aspects of high-quality maternal care remain under-recognized. Listening to patient’s perspectives during clinical care is a vital step to reducing care disparities and maternal morbidity, especially in underrepresented populations. Stressful birth events can increase the risk of psychological complications,^20,21^ particularly for those who do not receive adequate intraoperative and postoperative decision support, emotional support, or psychological follow-up care.^22^ These findings align with our previously unpublished observations from a related qualitative study of birthing people who experienced traumatic or emergent deliveries, particularly following an emergency team activation during birth. In that cohort, participants commonly described intense emotional responses—including fear, confusion, and long-term psychological distress—especially when information was not clearly communicated, or their birth preferences were not acknowledged. Many participants emphasized the importance of being informed in real-time, emotional validation, and empathic reassurance from care teams. These data reinforce our current findings that individuals undergoing intrapartum cesareans may require different preparatory and intraoperative communication strategies than those with scheduled procedures. Proactively managing expectations and clearly explaining deviations from the birth plan may mitigate the emotional impact of unplanned cesareans and improve overall patient experience. Although attending to physical needs during childbirth is critical, understanding patient perspectives on nonphysical priorities for cesarean delivery is also important.

The study findings also corroborate previous studies that support the importance of prenatal education for patient-centered outcomes. A 2013 study interviewed patients who had external cephalic versions to assess the impact of prenatal education on enhancing shared decision-making.^23^ Results showed differences between evidence-based information and lay beliefs, and that providers used various approaches to address these challenges. Similarly, our findings revealed that patients held lay beliefs about anesthesia side effects related to cesareans, underscoring the importance of providers offering clear and comprehensive information to support informed decision-making. Another interview study from 2021 found that while many pregnant people felt informed enough to decide on a planned cesarean, others felt pressured.^24^ Most patients we interviewed felt respected by providers and autonomous in their decisions based on provider recommendations. Some patient preferred preoperative discussions about potential scenarios in their delivery. It is important to note that these viewpoints are from patients who have already given birth, and the desire to hear about unpleasant intraoperative sensations and the detailed contingency planning may differ for those expecting vaginal birth. In reality, patients vary in the amount of detail they want in preoperative conversations, thus clinicians should be mindful of eliciting individual information needs and health literacy.

The theme of intraoperative pain aligns with existing studies on traumatic or complicated births, where pain is a devastating complication causing long-term physical and psychological distress. Interestingly, studies have shown that obstetricians and anesthesiologists often fail to reliably identify patient pain during surgery.^9^ Characterizing intraoperative discomfort is challenging due to varied sensations such as pressure and tugging as well as individual differences. A main difference between the groups in the current study was that providers expected patients to distinguish between pain and pressure, while many patients found this difficult, with some pressure sensations being painful to them. This discrepancy suggests that this pain versus pressure idea is rather unsuccessful, and the question should be, “Is this uncomfortable for you?” Prolonged pressure should be treated like pain, and pressure sensations should not be dismissed. Further studies on predictive factors of intraoperative pain^25^ and better pain assessment methods are needed. Accurate identification and immediate response to intraoperative pain are vital.^26^ Moreover, developing shared decision-making guidelines for intraoperative interventions on pain may be more important than identifying the exact sensation.

Patient interviews suggested that actions of health care personnel, such as providing emotional support, significantly impact perceptions of the birth experience, and provider interviews show their understanding of this impact. This finding can shape future research and quality improvement. Some patients reported discriminatory experiences during their hospital stay. Incorporating respectful maternity care into implicit bias training can improve the culture in the labor and delivery suite. Providers sensed that the hospital system and health care were strained, suggesting that more flexible protocols could better accommodate patient comfort. Additionally, improved coordination between obstetricians and anesthesiologists is needed to provide consistent information about pain during and after labor. Enhanced interdisciplinary collaboration can prevent discrepancies and gaps in informing the patient.

Social judgment and shame surrounding cesarean delivery warrant further exploration. Interviews with pregnant individuals who felt judged for having a cesarean delivery revealed a deep sense of stigma. Raising awareness about the medical necessity and safety of cesarean deliveries, supporting “natural” experiences such as skin-to-skin contact and breastfeeding, and empowering individuals regardless of mode of delivery are essential. Better awareness can reduce fear for those facing potential cesarean deliveries. Additionally, normalizing diverse birth experiences and emphasizing that every birth is unique and valid can be helpful. Providers play a pivotal role in influencing perceptions, and using inclusive language and avoiding judgmental attitudes can create a more supportive environment for those considering cesarean delivery.

Our secondary analysis of provider interviews by professional role revealed meaningful, albeit preliminary, distinctions. Anesthesiologists emphasized both the clinical complexity and emotional repercussions of intraoperative anesthesia experiences. Nurses focused on emotional support and continuous communication with patients during surgery, while obstetricians often reflected on how to honor patient autonomy amid surgical exigencies. These differences underscore the importance of considering role-specific perspectives when developing interdisciplinary strategies to enhance cesarean delivery experiences. Although our sample size precludes definitive conclusions, this subgroup analysis reinforces the need for further qualitative work exploring how team members’ roles shape their priorities and interactions with patients.

This study has several limitations. It included both scheduled and intrapartum cesarean deliveries, potentially masking key differences between these populations. Likewise, provider perspectives were clustered into one group, which may overlook theme differences between types of providers. Future studies may explore provider perspectives and label the provider type, distinguishing concerns between provider types. Data collection was conducted at two U.S. urban academic hospitals, limiting generalizability to nonurban centers or hospitals with fewer subspecialist staff. Although most patient interviews were completed within 72 hours of birth, recall bias and postpartum pain may have affected patients’ ability to accurately describe their experiences. It is also possible that other postpartum experiences were missed due to interviewing in the immediate postpartum period. Interviews were conducted only in English, excluding non-English-speaking patients and potentially missing important perspectives from minoritized populations. Similarly, majority of interviewed patients were educated, White, or both, constraining the present study’s conclusions to this cultural context. Studies in the future may purposively sample from vulnerable populations for better inclusivity.

Although interviews were limited to English-speaking patients and primarily reflected experiences at two urban, academic centers, the insights from this study offer important preliminary data to inform broader investigations. Future studies should include non-English-speaking participants and purposively sample more diverse populations to enhance inclusivity and representativeness

## Conclusions

Although both patients and providers value pain management, psychological well-being, and communication in cesarean delivery care, their perspectives differ in some respects. Patients emphasize trust and emotional support, while providers focus on clinical strategies. Bridging these gaps through tailored education and shared decision-making may enhance patient experiences and provider–patient alignment.

## Data Availability

The datasets used and/or analyzed during the current study are available from the corresponding author on reasonable request.

## Abbreviations Used

BMI: body mass index
CNM: certified nursing midwife
CRNA: certified registered nurse anesthetist
NICU: neonatal intensive care unit
no.: number
PA-C: physician-assistant certified
