# Supplementary material for: Patient and Provider Perspectives on Cesarean Delivery Pain and Anesthesia Experiences: A Qualitative Study
Source: Womens Health Rep (New Rochelle). 2025 Aug 1;6(1):711–22. doi: 10.1177/26884844251364123 (PMC12413247; doi:10.1177/26884844251364123)
Supplement: Supplementary Data S1 [file 26884844251364123_supplementary_data_s1.docx]

**Additional file 1.** Semi-structured interview guide.

***Patient group***

**1. Attitudes, Beliefs and Narrative**

- To start, could you tell me about your experience with your most recent cesarean delivery?
  - (Note to interviewer – be sure to cover ground related to their perceptions: What things did they think went well, what things could have been better)
- Did your experience match what you were expecting would happen for your cesarean?
  - If no – Can you tell me more about that?
  - If yes, move on.
- Some people find that being pregnant, or female, or in a minoritized group, or other characteristics, can affect their overall birth experiences and the way people are treated during and after birth. Do you believe that any of these characteristics, impacted your cesarean delivery experiences, positively or negatively?
  - If so, how?

**2. Expectations about cesarean pain and its management**

- Can you tell me about what you were expecting about pain during and after birth?
  - Did your provider talk to you about pain management options? If so, what options?
  - Did you do anything to prepare yourself for pain related to childbirth? If so, what things did you do? Do you think preparations helped you deal with pain?
- Some people experience pain during their cesarean birth. If you were to experience pain, ideally, what kinds of options would you want to help you manage pain during or after birth?
- Some treatments for pain or discomfort, can cause side effects like sedation, amnesia (forgetting events and experiences that are happening around you) nausea, vomiting, itchiness, or shivering. How important are treatment side effects to you, in making decisions about how you would want to treat your pain or discomfort during your cesarean birth?
- Did you experience pain during your cesarean delivery?
  - If yes – Would you like to tell me about that experience? (note to interviewer – be sure to cover their perceptions on what options they had to treat it, how freely they felt they could communicate with the anesthesiologist, or their priorities regarding their treatment vs. the experience of pain)
  - If no – move on to next sections

**3. Respectful maternity care**

- How important is it to you, to feel respected during and after your birth?
- In what ways would you feel like you had been treated respectfully?
- What do you think are the most important things for clinicians to know about when it comes to accomplishing goals of respectful and dignified birth?
- Some people consider certain elements to be essential parts of respectful and dignified birth care. These elements include things like: clinician verbal attitudes and conversation to each other, providing explanations to patients for all procedures and events in real time, and ready access to and compassionate communication from clinicians. What do you think about that?

4. **Shared Decision Making**

- How important is it to you, to feel like you have full control over decisions during childbirth?
- Some people think that feeling free from pressure to accept interventions, and feeling heard are important parts of their birth experience. What do you think about that?
- Consider the conversations you had during your care so far, that involved making decisions about your care. Did you feel you received timely communication from your care team? Did you feel the information was high-quality? How important are these things, to you?

5. **Emotional Support**

- Some people have had difficult birth experiences. If you had that, how important to you would it be to be able to talk about their experience with a care team member? When would you want to have that conversation?

**6. Closing**

- Is there anything else you would like to add that we did not discuss?

## ***Anesthesiologist Group***

**1. Attitudes, Believes, and Narrative**

- From your point of view, what are the most important outcomes for cesarean delivery?
- What do you think patients believe are the most important outcomes for cesarean delivery?
- Some people think that pregnancy, being female, or from a minoritized group, can affect their overall birth experiences and their clinical care during birth. Do you think these characteristics affect cesarean delivery experiences, positively or negatively? If so, how?
- Some patients experience pain or discomfort during cesarean delivery. Do you routinely discuss pain management options and plans around these possibilities with your patients? Tell me more about that.
- Do you believe patients are well prepared for the possibility of pain during cesarean delivery, and their options for treating it? Tell me more about that.
- What do you think are the most important things for clinicians to know about when it comes to pain during cesarean delivery?

## **2. Respectful maternity care**

- From your point of view, how important is respectful maternity care in anesthesia practice? In what ways do you intentionally support dignity and respect during cesarean delivery?
- What do you think are the most important things for clinicians to know about when it comes to accomplishing goals of respectful and dignified birth?
- Some people consider certain elements to be essential parts of respectful and dignified birth care. These elements include things like: clinician verbal attitudes and conversation to each other, providing explanations to patients for all procedures and events in real time, and ready access to and compassionate communication from clinicians. What do you think about that?

3. **Shared decision making**

- How do you support decision making with patients having cesarean delivery?
- What do you think are the most important things for clinicians to know about when it comes to accomplishing goals of shared decision making?

4. **Emotional support**

- How important do you think it is to provide emotional support during and after birth for people who had a difficult birth? What do you do to provide that in your practice?

**3. Closing**

- Is there anything else you would like to add that we did not discuss?

## ***Labor and delivery nurse group***

**1. Attitudes, Believes, and Narrative**

- From your point of view, what are the most important outcomes for cesarean delivery?
- What do you think patients believe are the most important outcomes for cesarean delivery?
- Some people think that pregnancy, being female, or from a minoritized group, can affect their overall birth experiences and their clinical care during birth. Do you think these characteristics affect cesarean delivery experiences, positively or negatively? If so, how?
- Some patients experience pain or discomfort during cesarean delivery. Do you routinely discuss these things with your patients? Tell me more about that.
- Do you believe patients are well prepared for the possibility of pain during cesarean delivery, and their options for treating it? Tell me more about that.
- What do you think are the most important things for clinicians to know about when it comes to pain during cesarean delivery?

## **2. Respectful maternity care**

- From your point of view, how important is respectful maternity care during cesarean delivery? In what ways do you intentionally support dignity and respect during cesarean delivery?
- What do you think are the most important things for clinicians to know about when it comes to accomplishing goals of respectful and dignified birth?
- Some people consider certain elements to be essential parts of respectful and dignified birth care. These elements include things like: clinician verbal attitudes and conversation to each other, providing explanations to patients for all procedures and events in real time, and ready access to and compassionate communication from clinicians. What do you think about that?

3. **Shared decision making**

- How do you support decision making with patients having cesarean delivery?
- What do you think are the most important things for clinicians to know about when it comes to accomplishing goals of shared decision making?

4. **Emotional support**

- How important do you think it is to provide emotional support during and after birth for people who had a difficult birth? What do you do to provide that in your practice?

**3. Closing**

- Is there anything else you would like to add that we did not discuss?

***Obstetrician / Midwife Group***

**1. Attitudes, Believes, and Narrative**

- From your point of view, what are the most important outcomes for cesarean delivery?
  - What are some things that as an obstetrician, you prioritize when caring for a patient throughout a c-section from pre-op to post-op, and following up even past that?
- (On the flip side), what do you think patients believe are the most important outcomes for cesarean delivery?
- Some people think that pregnancy, being female, or from a minoritized group, can affect their overall birth experiences and their clinical care during birth. Do you think these characteristics affect cesarean delivery experiences, positively or negatively? If so, how?
- Some patients experience pain or discomfort during cesarean delivery. Do you routinely discuss these things with your patients? Tell me more about that.
- Do you believe patients are well prepared for the possibility of pain during cesarean delivery, and their options for treating it? Tell me more about that.
- What do you think are the most important things for clinicians to know about when it comes to pain during cesarean delivery?

## **2. Respectful maternity care**

- From your point of view, how important is respectful maternity care during cesarean delivery? In what ways do you intentionally support dignity and respect during cesarean delivery?
- What do you think are the most important things for clinicians to know about when it comes to accomplishing goals of respectful and dignified birth?
- Some people consider certain elements to be essential parts of respectful and dignified birth care. These elements include things like: clinician verbal attitudes and conversation to each other, providing explanations to patients for all procedures and events in real time, and ready access to and compassionate communication from clinicians. What do you think about that?

3. **Shared decision making**

- How do you support decision making with patients having cesarean delivery?
- What do you think are the most important things for clinicians to know about when it comes to accomplishing goals of shared decision making?

4. **Emotional support**

- How important do you think it is to provide emotional support during and after birth for people who had a difficult birth? What do you do to provide that in your practice?

**3. Closing**

- Is there anything else you would like to add that we did not discuss?
