# Supplementary material for: Patient and Provider Perspectives on Cesarean Delivery Pain and Anesthesia Experiences: A Qualitative Study
Source: Womens Health Rep (New Rochelle). 2025 Aug 1;6(1):711–22. doi: 10.1177/26884844251364123 (PMC12413247; doi:10.1177/26884844251364123)
Supplement: Supplementary Data S2 [file 26884844251364123_supplementary_data_s2.docx]

**Additional file 2.** Detailed quotations for patient and provider themes. Themes were identified by semi-structured interviews with patients with lived experience with cesarean delivery, and providers who provide cesarean delivery clinical care.

| ***Patient Perspectives*** | | |
| --- | --- | --- |
| ***Theme*** | ***Code*** | ***Quotation*** |
| **Effective communication, education, and respect** | Desire to understand back-up plan or alternative options in case of unexpected events | “I’m a planner, I need to understand. Yeah, this is great that we have this beautiful plan ahead of us, but what if that plan doesn’t work out? I need to know what you’re going to do so I’m prepared for it mentally.” |
|  | Importance of tailoring education to patient’s level of medical literacy | “Everybody has a different capacity for understanding things and… the things the providers say should be accessible to everyone.” |
|  | Real-time updates during procedure made patient feel comfortable | “…there were nurses everywhere and. They were keeping me informed, calming me down…”  “They talked to me the whole time. I talked to them, and it was just more of a pleasant experience… it wasn’t scary.” |
|  | Perceived discriminatory experiences while receiving care | “I think my weight… it probably added complications that there wouldn’t have been otherwise.”  “I do feel like me being 20 years old, some doctors looked at me a little bit different… the nurse was very judgy… I do think when it comes to parenting at a young age, I do feel like it is very judgy from other people’s perspectives.” |
| **Emotional support by care team** | Mental/emotional effect of being awake during a major surgery | “Mentally, I think it’s a little bit more tough to take because the whole thing for me is, you’re going into a surgery and you’re wide awake and they’re moving around organs and taking things out. And you’re like, oh my goodness, I am awake for this.” |
|  | Importance of mutual respect and its effect on building trust | “Mutual respect is everything. If you feel like you’re not being respected then I don’t trust you have my best intentions at heart and you’re going to be cutting into my abdomen and I don’t feel like you’re, I wouldn’t feel like you’re listening to me or handling my concerns, so that’s huge.” |
|  | Care team created a comfortable environment for patient | “I was fully tense and I was just crying. And everyone over there, they just boosted me up. They gave me the strength to go for the operation…” |
| **Intraoperative pain or discomfort** | Patient felt uncomfortable (but not in pain) during cesarean delivery due to unexpected sensations | “It was just the tugging that I felt… It was getting too much and it was making me not queasy… I want this to stop… it’s hard to explain…” |
|  | Patient was uncomfortable during cesarean delivery and felt pain | “I don’t think I was completely numb because I was in a lot of pain when it came to the time of when they pulled her out and I felt every single amount of pain the whole entire time at that point.”  "I will say I did kind of feel a little bit. I don’t think I was completely numb because I was in a lot of pain when it came to the time of when they pulled her out and like I felt every single amount of pain the whole entire time at that point. I ended up, I guess from what my fiancé said, passing out for a minute and then waking right back up. I did throw up during it a couple I think once or twice. But I was so exhausted. I mean, I pushed for five hours prior to having a C-section. So other than that, I mean it went OK." |
|  | Inadequate intraoperative pain management by care team | "I woke up... saying I feel pain and that the nurses and whoever else is in the room with me were like you shouldn't be feeling that, you shouldn’t be feeling that. And it was too late. They gave me more heavy medications to take the pain away, but it was too late. She was already born." |
|  | Pain management options were not discussed with patient | "I don't think they...they talked to me about options they told me I would be given this… I was under the assumption that's exactly what is given. |
|  | Patient fear of intraoperative pain | “It was a fear that what if the spinal doesn't work and I start feeling things, but all was well there, thank God. But I've heard instances of women not being believed that. Oh, don't worry, it's still kicking in, but it had completely stopped. And then they felt everything. So that was like a nightmare in my mind but everything was fine."  "That was my biggest concern; was not feeling anything during the procedure, and we may get into this more with your questions, but it was quite a bit different from having an epidural and going into a C-section, I would say, than having a spinal and experiencing that. Very different.” |
|  | Patient felt concerns regarding intraoperative pain were heard by anesthesiologist | "Some people experienced pain during their cesarean. If you were to experience that ideally what kind of options would you want to help you manage that?”  “I actually did experience pain during my c-section and they were very like, I don't want to say insistent because it was coming from a place of wanting to help. It was just like if you're feeling anything, tell us. And so I was very clear, like, hey, I'm feeling like a sharp pain right now. And they were really good about giving me pain medicine in that moment before they would proceed any further.” |
| **Varying acceptability around pain therapies** | Patient wishes to avoid opioids due to side effects | “I didn’t want any [opioids] because I just never had narcotic… I mean what you think you know about those, I don’t want to be taking care of a baby while I’m on a narcotic, so it’s weird. I don’t want to touch that.”  “…I just feel like [opioids] is like next level… that’s like people could get addicted to that if you know what I mean… So I just, I don’t want to mess with that.” |
|  | Patient concerns surrounding anesthesia side effects | “…when I hear side effects, I get more concerned about the long-lasting ones… You hear with an epidural you could have chronic migraines.”  “I didn’t want an epidural… I was scared of getting those medication headaches, swelling, shaking, that kind of stuff.” |
|  | Patient would prefer general anesthesia | "I honestly did not expect to feel any of it. I didn't feel them cutting into me or anything, but when the part came to deliver her and when she came out, I didn't expect to actually feel that. When it came time for her to officially come out and be delivered, I didn't expect to feel all that amount of pain that I felt. And to me personally, I would’ve felt better being put under than to be just drugged up.” |
|  | Patient prioritized baby/baby's needs over pain management | "I'm the type of person that will be thinking about that so you're almost kind of amped up a little bit in a way. And so if you did feel pain, that's why I'm glad, [the anesthesiologist] told me what the plan was going to be for that, because if I'm experiencing that, I know what the team is going to do to take care of situation, and I would want... First and foremost, I would want whatever was best for my son to happen. If you get pain meds, it goes right to him, you know? So if he wasn't born yet and that was happening, I would deal with whatever I had to, to make sure he was here and okay. And then give me the most pain meds that you possibly can. But the top priority would be with my son and just trying to bear through that until he was safe and sound." |
| **Stigma surrounding cesarean delivery** | Efforts or desire to mirror vaginal birth experience as best as possible. | “I was really appreciative that they were able to delay the cord clamping for three minutes instead of the standard one minute… they were able to honor the skin-to-skin and for the most part, my baby was with me and her cheek was to my cheek, which made it really special… So a lot of what I had hoped for for the natural birth experience, they were able to accommodate, which was very meaningful to experience.”  “It was just more of me feeling like part of womanhood is like, you have to birth my baby, and I was worried that the cesarean and it being so clinical and surgical wouldn’t feel like that experience of giving birth that I was hoping for and anticipating when going down this journey… So it’s been an internalized identity crisis.” |
|  | Social judgment surrounding birth and motherhood | “What I actually think is one of the most disheartening things is that women to other women will make comparisons about whether you have a natural birth or as my mother-in-law calls it, a “real” birth, versus a c-section. And I think both of those are real births, that one is not less than the other… We do it with breastfeeding. If you don’t breastfeed, you know women are really hard on each other about it.” |
| ***Provider Perspectives*** | | |
| ***Theme*** | ***Code*** | ***Quotation*** |
| **Complexity of pain responses** | Recognized complexity of pain responses and expressions. | “Not all women have the same pain tolerances. They don’t have the same past histories. They don’t have the same subjective experiences. A lot of the times, the way you experience something is based on just your entire lifetime of experiences… So pain – I feel like it’s important that people understand the pain is not just about the medicines we’re giving… but it’s all other cultural and emotional aspects of pain… It’s very subjective.” |
|  | Importance of individualized pain management plans. | “You’ll have a c-section where the person feels nothing from start to finish, and you’ll do the exact same anesthesia care for somebody else and they will have a lot of issues with being comfortable… So knowing that one treatment or one recipe does not fit all and that you have to individualize your approach.”  “It’s important to know that every patient is different and every situation is completely unique and the responses to the medications and epidurals and regional anesthetic are very unique and it shouldn’t be treated the same.” |
| **Multiple pain control strategies** | Importance of patient education on variety of postoperative pain management methods. | “When patients have reasons for going beyond our standard therapy, we do acute pain blocks. Tap blocks we do to help with post-op pain and patients are candidates for it because they may have OUD or can’t get opioids for one reason whether it’s an allergy…” |
|  | Importance of multimodal pain management. | “Multi-modal analgesia is a big part of cesarean analgesia. So to make sure that whether it’s intra-op or post-op, that the patient is getting everything from that multi-modal arsenal that they can, because all of them do work in a complementary way.” |
| **Effective communication during emergency cesarean delivery** | Desire for better education on intraoperative pain management despite emergency setting | “In an emergency c-section, I feel like sometimes they might not be quite as well informed as a scheduled… when things happen so quickly and we have to do a general anesthetic that they’re not fully aware that they are going to be in a decent amount of pain…” |
|  | Perceives education reduces patient anxiety | “I think a lot of people are anxious because of the unknown. It’s things they do not know and they are anticipating that are going to happen… So maybe a priority would be being informed on step by step what’s going to happen from start to finish and having a better idea of what they should expect.” |
|  | Balance between provider opinion and patient autonomy | “Almost always there's more than one option. There's more than one right way… Just the other day. I was over at Magee and there was someone who had been pushing awhile and the doctor recommended C-section and the patient asked can I push one more hour and that was a totally reasonable request, you know? And that doctor granted it. And then still had a safe cesarean an hour later, and I realized I'm not a surgeon, and they have a really hard job. So I acknowledge that, but almost always, there's options. And we can give the patients all the information. We might be able to say, “This is maybe what I would do in your shoes if you want to.” But again, back to the patriarchy, we are still in this pattern of you need the C-section, we just have to go do this… Give them all the options that you possibly can that are within reason, and then support whatever informed decision they make. People make a lot of different decisions and a lot of them are reasonable.”  “I think shared decision-making in some places, but in other places, the patients really don't have the expertise. I was giving you an example of the breech deliveries, and there have been other times too when the obstetricians I guess gave the patient too much leeway, like a prolonged labor, and they're pushing and pushing the patient and the obstetrician just knows it's never going to work. And I think then the woman can become infected and all that kind of stuff and have more problems, you know? And that's where the obstetrician really kind of needs to be firm. I mean, that's their expertise.” |
| **Patient psychological well-being during cesarean delivery** | Priority for respectful communication and trauma-informed care | “…patients may have had a traumatic past birth. So I think just being respectful of how they are; they may be super anxious, it might be a PTSD trigger from what they experienced before.”  “Number one, everyone should use trauma informed care at every interaction with anybody, ever. So trauma informed care should be the standard. There should be permission asking and patients should know that they can stop anything at any time. So I think centering trauma informed care, because at least 1 in 4 people come into our healthcare system already experiencing some kind of sexual assault history…”  “If there happens to be any initial evaluation of potential birth trauma even identified immediately after delivery or in the immediate postpartum period, we do have a good set of resources, not only from the inpatient social work side, but then also a subset of different resources that we include on their discharge, paperwork. If at that time they're not in the head space or feel the need to have those resources, we still provide…hey, these are different either support groups or therapists or things of that nature… And then we also do a EPDS on every patient, prior to discharge...So then we have our first kind of initial screening from that standpoint too in order to screen patients that might be at higher risk for postpartum depression and make sure that they have a short interval follow up schedule.” |
|  | Perceives patient trauma with general anesthesia | “…they were in a lot of pain and then they went to sleep and now there’s a time in their life, maybe arguably one of the most important times in their life, and they can’t remember it. That’s some real trauma.” |
| **Barriers to observing the patients’ birth plans** | Desire to increase flexibility and avoid rigidity of protocols | “…certain providers actually didn’t mind turning off the lights. They would just keep the spotlights on… [turning them off would have] positively affected patients…”  “Knowing that there’s a lot of rules in an OR, knowing that there are a lot of things that are not up to me nor the patient…” |
|  | Bonding with newborn | “…there are certain situations where a patient has come in and wants to do skin-to-skin and then they’re throwing up the entire time during the c-section. So then it’s kind of like, well, we can’t put the baby on your chest while you’re actively vomiting. So I think that that is hard even for me because I want them to be happy and enjoy this experience as best as they can…”  “Sometimes patients will say, I don’t know if I can hold the baby right now. Like I’m shaking or I feel unsafe. I always reinforce that the shaking would be happening even if they were having a vaginal birth…” |
|  | Acknowledging “beauty of birth” beyond medical standpoint | “Magee, it’s such a machine… we do c-sections and epidurals all day. So you kind of feel like oh, they’re just another patient, but for this patient, this is their whole world. They’re birthing a child… it’s their body, it’s their child, it’s their birthing experience that they’ll talk about for years and years, good or bad. So just being really cognizant that you’re really in a monumental place in their life. So to just try to act the best way you can.”  “I think a lot of times when you’ve been doing it for so long, these seem like such a routine, normal procedure for us, and it’s hard to take a step back and just look at the whole overall picture of how this person’s life is about to completely change… I think sometimes we get into the mindset of we need to get this procedure done, we have another one waiting… instead of sometimes giving them the time that they need to really go over everything with them or talk to them afterwards.” |
|  | Perceives challenges to supporting provider gender preferences | “…with the females preferred, that’s the one that just affects us the most. Sometimes it can be a little bit frustrating because we don't have the same values or views on those issues, and you're in the tough spot where you don't have a lot of providers and accommodating those sometimes can be hard and put stress on the team. But being able to go beyond what your views are and saying, hey that patient has a certain set of wishes and how can you try to make it happen? …I mean, this is their birth experience. Let's try to respect those wishes.” |
|  | Views healthcare system as overburdened or overstressed | “We should be flexible and allow those things if that's what's important to the patient and it's not a safety issue, but I think people sometimes are...So just like I don't know if it's burnout or they don't have the time or they're so fixated on just getting through the day and getting to the procedures that they forget that that's important.” |
